# Psychological experience of patients with confirmed COVID-19 at the initial stage of pandemic in Wuhan, China: a qualitative study

**DOI:** 10.1186/s12889-021-12277-4

**Published:** 2021-12-11

**Authors:** Tiantian Li, Yingjie Hu, Lei Xia, Lihua Wen, Wei Ren, Wei Xia, Jia Wang, Wenzhi Cai, Ling Chen

**Affiliations:** 1 Department of Nursing, Shenzhen Hospital, Southern Medical University, Number 1333, Xinhu Road, Baoán District, Shenzhen, 518101 China; 2 School of Nursing, Southern Medical University, Guangzhou, 510515 China; 3 Department of Traumatology, Tongji Hospital affiliated to Tongji Medical College, Huazhong University of Science and Technology, Wuhan, 430030 China; 4 Department of Hepatobiliary, Pancreatic and Splenic Surgery, Suzhou Ninth People’s Hospital, Suzhou, 215299 China; 5 School of Nursing, The University of Hong Kong, Hong Kong, 999077 SAR China

**Keywords:** Coronavirus disease 2019 (COVID-19), Infectious disease pandemic, Patients, Psychology, Qualitative research

## Abstract

**Background:**

Coronavirus disease 2019 (COVID-19) quickly developed into a global pandemic and affected patients’ mental health. However, little is known about psychological experience of patients with COVID-19. The aim was to elucidate the psychological experience of patients with confirmed COVID-19 in Wuhan, at the initial stage of the pandemic.

**Methods:**

This study was conducted using a phenomenological approach in a qualitative study. Thirteen patients with confirmed COVID-19 from a COVID-19-designated hospital in Wuhan, were recruited between March 15th and April 20th, 2020 via purposive sampling. Semi-structured in-depth interviews were conducted face-to-face. The interview data were analyzed using inductive thematic analysis.

**Results:**

The psychological experience of patients was summarized into three themes: mental distress related to COVID-19, expectations of life scenarios after discharge, and making sense of the experience. These themes were classified into 10 sub-themes. Patients experienced confusion, uncertainty, worry, guilt and concern. Both positive and negative expectations of life scenarios after discharge were reported, manifested as expectations about making up for lost time with family, anxiety about social discrimination and feelings of helplessness about poor financial security. Moreover, patients perceived strength of abundant social support and awareness of social responsibility from their unique experience to cope with their condition.

**Conclusions:**

This study demonstrated that patients with confirmed COVID-19 in Wuhan underwent complex psychological experience, both positive and negative at the initial stage of the pandemic. These findings will contribute to the delivery of effective mental health care to safeguard patients’ wellbeing.

## Background

A cluster of pneumonia cases caused by severe acute respiratory syndrome coronavirus 2 (SARS-CoV-2) were reported in Wuhan, China, at the end of 2019 [1, 2].The resulting disease was officially named coronavirus disease 2019 (COVID-19) by the World Health Organization (WHO) [3]. COVID-19 is mainly transmitted through respiratory droplets and close contacts [4], and rapidly spread worldwide. On March 11, 2020, the WHO officially declared that COVID-19 was a pandemic [5].

Throughout history, the outbreak of acute respiratory infectious diseases have lead to mental disorders among patients [6]. For example, anxiety and fear were the most common psychological responses observed during previous pandemics of severe acute respiratory syndrome (SARS), influenza A (H1N1), and Ebola; other manifestations, such as depression and posttraumatic stress disorder also occurred [7]. Similar to other epidemics, COVID-19 pandemic not only brings about physical sufferings but also leads to a series of psychological problems [8]. The patients may suffer from anxiety, depression, fear, panic posttraumatic and stress symptoms, [6, 9]. Each of these symptoms has adverse effects on the recovery process of the disease [10]. This highlights the increasing need to pay attention to mental health of patients during the disease outbreaks.

However, at the initial stage of the COVID-19 pandemic, WHO and other health authorities were mainly focused on exploring the epidemiology, transmission patterns, clinical features, and treatment of COVID-19 with little knowledge on the psychological impact of patients [11], especially in the hard-hit areas in China. Existing studies on the scope of psychological dimension have typically focused on the health care workers (HCWs), social vulnerable groups [12], which calls for further research aimed at investigating the psychological experience of patients. It will inform the mental healthcare systems’ responses to protect the mental condition of patients.

Therefore, the current study sought to qualitatively investigate the psychological experience of patients confirmed with COVID-19 in Wuhan at the initial stage of the pandemic. Due to the unique, complexity and multi-dimension of individuals’ lived experience and their inner world, the method of qualitative research is appropriate and offers the opportunity to gain a deeper understanding of COVID-19 patients’ psychological experience [13]. The results of this research could therefore greatly contribute to deliver the better psychological care and handle the disease more effectively.

## Methods

### Design

A phenomenological approach was adopted in a qualitative study to elucidate the psychological experience of patients with confirmed COVID-19 from the perspective of the patients [14]. The current study followed the consolidated criteria for reporting qualitative research guidelines [15].

### Recruitment

Participants were recruited through purposive sampling from a COVID-19-designated hospital in Wuhan, between March 15th and April 20th, 2020. Nurses (L.H.W.and L.X.) delivering nursing care for patients with COVID-19 performed the recruitment. Given that participants should be knowledgeable on the research question and can articulate [16], the inclusion criteria included: (1) age ≥ 18 years old; (2) meeting the diagnostic criteria for COVID-19 according to the Diagnosis and Treatment Guideline for Patients with Novel Coronavirus Pneumonia (5th trial version) [17]; (3) without language barriers. Patients who were emotionally unstable, had difficulty communicating, or were critically ill, were excluded. After selecting according to the inclusion criteria and exlusion criteria, purposeful sampling was used, which is one of the major differences between qualitative and quantitative research approaches [16]. By utilizing purposive sampling with maximum variation sampling technique, participants’ demographic variation were reached and participants recruited before can provide direction for subsequent participant recruitment. More details of participant recruitment flow were shown in the Table 1. And as a result of purposive sampling, a heterogeneous sample of patients confirmed with COVID-19 were recruited, who varied in demographic characteristics, including age, sex, educational level, and employment to obtain comprehensive and diverse psychological experience of patients with COVID-19. The sample size was determined following the principle of data saturation that the subject data recurred and no new themes emerged during data analysis [16].

**Table 1:** Different demographics and clinical characteristics of each participants with the recruitment process (*n* = 13)

Participant	Age, years	Sex	Educational Level	Occupation
P1	64	Male	Junior high school	Farmer
P2	59	Female	Senior high school	Unemployed
P3	44	Female	Senior high school	Labourer
P4	57	Female	Junior high school	Cleaner
P5	43	Female	Junior high school	Farmer
P6	38	Male	Bachelor	Company employee
P7	36	Male	Bachelor	Company employee
P8	57	Male	Junior high school	Unemployed
P9	52	Male	Junior high school	Farmer
P10	33	Male	Master	Company employee
P11	30	Male	Bachelor	Company employee
P12	30	Female	Master	Company employee
P13	31	Female	Bachelor	Company employee

### Participants

Data saturation was reached with 13 interviews which was jointly decided by the research team. The participants consisted of seven males and six females. They have not been included in other reports. According to Diagnosis and Treatment Guideline for Patients with Novel Coronavirus Pneumonia (5th trial version) in Chinese [17], clinical classification of all participants were common type which referred to patients with fever, respiratory tract and other symptoms with pneumonia in imaging. These participants received treatment including antiviral therapy, oxygen therapy, illness monitoring, and other supportive therapy, such as enough rest, maintaining nutrient balance and internal environment stability. There were two patients with other comorbidities, with one was hypertension and the another was diabetes mellitus. Six patients’ SARS-CoV-2 virus nucleic acid test on nasopharyngeal swabs revealed a re-detectable positive result after negative detection. There was only one patient declining to participate in this study due to unwillingness to undergo the interview and complete the questionnaire. Table 2 summarized the demographics and clinical characteristics of these participants.

**Table 2:** Demographics and clinical characteristics of participants (*n* = 13)

Variables	Characteristics	*n* (%)
Age, years	30–39	6 (46.2%)
	40–49	2 (15.4%)
	50–59	4 (30.8%)
	60–69	1 (7.7%)
Sex	Male	7 (53.8%)
	Female	6 (46.2%)
Nationality	Han Chinese	13 (100.0%)
Marital status	Married	13(100.0%)
Educational level	Junior high school	5 (38.5%)
	Senior high school	2 (15.4%)
	Bachelor	4 (30.8%)
	Master	2 (15.4%)
Occupation	Employed	11 (84.6%)
	Unemployed	2 (15.4%)
Clinical classification^a^	Common	13 (100.0%)
Comorbidities	Hypertension	1 (7.7%)
	Diabetes mellitus	1 (7.7%)
	No	11 (84.6%)
Re-detectable positive^b^	Yes	6 (46.2%)
	No	7 (53.8%)

^a^ As to clinical classification, common type referred to patients with fever, respiratory tract and other symptoms with pneumonia in imaging based on Diagnosis and Treatment Guideline for Patients with Novel Coronavirus Pneumonia (5th trial version) in Chinese [17]

^b^ “Yes” referred to that a re-detectable positive result from SARS-CoV-2 virus nucleic acid test on nasopharyngeal swabs occurred in those patients after negative detection. “No” referred to the SARS-CoV-2 virus nucleic acid test on nasopharyngeal swabs of those patients were always positive

### Data collection

Data were collected via in-depth face-to-face interviews to analyze the psychological experience of patients with COVID-19. Interviews involved interactions between the interviewers and interviewees based on interview questions, that researchers pose questions and the participants provide answers [16]. An interview guide was needed to develop beforehand and identify the questions what would be asked in the interview. Through literature review and expert consultation, a semi-structured interview guide was jointly developed by all members of the research team to ensure the appropriateness of it. The purpose of literature review and expert consultation was to create a appropriate, clear, comprehensive, focused interview guide by seeking researchers’ and experts’ opinions. It was helpful to determine essential questions in relation to the research aim [18]. Besides,the interview guide was piloted with three patients to refine it. Interview data collected during the pilot study were excluded from the final analysis. The final interview guide consisted of two parts: open-ended questions and probe questions (Table 3).

**Table 3:** Semi-structured interview guide

Open-ended questions	Probe questions
What is your experience as a patient with COVID-19 in hardest-hit area of China during this outbreak?	Comfortable/uncomfortable?
How does the outbreak of COVID-19 impact upon you?	Challenges/opportunities?
Is there anything else you would like to share with me concerning the COVID-19 outbreak?	Pleasant/unpleasant?

Note: *COVID-19* Coronavirus disease 2019

The interviews were performed by the researchers (L.H.W. and L.X.), who were frontline nurses with formal training in qualitative study and interview methods. Patients were invited to participate in the interview after an explanation of the objective and procedure of this study. After expressing willingness to participate the interview, written informed consent was obtained from patients. Subsequently, a convenient time was arranged for the interviews. To avoid external interference, the interviewers (L.H.W.and L.X.) conducted in-depth face-to-face interviews in a quiet, interference-free, separate ward without any others that met the criteria for pandemic prevention and control. Each interview was guided by a standardized procedure (introduction, interview, and end). Before starting the interview, participants firstly completed a questionnaire survey about their age, sex, nationality, marital status, educational level, occupation, clinical classification of COVID-19, comorbidities and whether virus nucleic acid test on nasopharyngeal swabs revealed a re-detectable positive result after negative detection. Participants were then invited to freely express their feelings and experience. The order of the questions asked was flexibly adjusted according to the specific situation during the interview. Meanwhile, any hints or inducements were avoided. All interviews were audiotaped after gaining permission from participants. Non-verbal clues, such as facial expressions and emotional changes of participants, were observed during the interview and recorded in field notes. The unclear statements were also timely checked with participants through retelling and clarification during the interview to eliminate ambiguities. Participants were encouraged to elaborate their psychology experience, through the follow-up inquires, such as “Do you mean...?” “What do you mean?” and “Can you explain for that a bit more? All interviews were conducted between March 15th and April 20th, 2020 and lasted approximately 20–30 minutes, taking an average time of 25 minutes, which referred to the formal interview time in standardized procedure. It was worth noting that the formal interview time did not include introduction/end time, whereas questionnaire survey time did. The time of providing the interviewee with a series of information, both about the study and informed consent about their rights was performed before the interview and was not included in the average interview time of 25 minutes.

### Data analysis

Data collection, management and analysis were carried out concurrently. After the interview, audio recordings were transcribed verbatim into texts by researchers (T.T.L. and Y.J.H.) within 24 hours. After transcription of the audio recordings into text, the text was annotated with field notes containing non-verbal clues of participants to assist data analysis. Two researchers (T.T.L. and Y.J.H.) independently analyzed data manually. Inductive thematic analysis was adopted to obtain the data-driven results [19]. The first step was to repetitively read the transcript to immerse in the data, familiar with it and gain a full understanding of the interview data. The second step was to identify meaningful statements from phrases and sentences using a color-coding method to generate the initial codes. Through this step, all actual data extracts were coded. And then these meanings and initial codes were then extracted and placed into a table format to collate each code by analyzing the relevance of them. The third step was to search potential themes and sub-themes by combining all the relevant codes and data extracts into clusters and categories. The forth step was to review and refine themes by comparing the external heterogeneity between themes and internal homogeneity within the themes, including the level of coded data extracts and entire data as a whole. The fifth step was to define and name the themes to identify essence of them. The final step was to prepare the report through selecting vivid examples and relating them to the research question. The quote examples were reported in participants’ own words without any change to ensure the precision. Two authors (T.T.L. and Y.J.H.) reviewed and extensively discussed the themes and sub-themes in several meetings during the analysis process. Any discrepancies were discussed in research team meetings with all co-authors to reach consensus. With reference to the field notes, it assisted the verbal interview data analysis as an additional data source. Finally, a full description of observed phenomena was formulated.

### Rigor

Several strategies were adopted to ensure methodological rigor, credibility and trustworthiness. First, the interviewers (L.H.W.and L.X.) were frontline nurses delivering services for patients with COVID-19 in Wuhan and established mutual trust with participants through prolonged engagement before the interviews. Second, triangulation was performed to collect and analyze data. Data from multiple sources (field notes and audio recordings) were analyzed independently by two researchers (T.T.L and Y.J.H). The newly emerging themes were repeatedly checked against the original transcribed text to ensure that the data analysis and interpretation were driven by the subjects. Third, meetings were held regularly among the members of the research team for peer debriefings. The data analysis results were compared and discussed until reaching consensus to increase the reliability and accuracy of the results. Fourth, audit trails were used to ensure that the data collection and analysis steps could be traced to the original interviews.

### Reflectivity in the qualitative study

Reflectivity is an integral part of qualitative study, which can help recognize the preconceptions of the researchers and avoid the subjective influence of the researchers on the research results [20]. Three researchers in the research team have doctoral degrees in nursing science (W.X., W.Z.C. and L.C.), four researchers have master’s degrees in nursing science (T.T.L., Y.J.H., W.R. and J.W.), and two researchers have bachelor’s degrees in nursing science (L.X. and L.H.W.). All researchers are female and received formal training in qualitative study methods, either in school education or in continuing education. The members of the research team have engaged in clinical nursing and scientific research work for a long time, and have deep experience. During the fight against COVID-19, the research team, whose research field is rehabilitation medicine, has been dedicated to psychological rehabilitation of patients with COVID-19 and frontline HCWs. To avoid the influence of researchers’ personal experience, views, and preconceptions on the analysis and interpretation of the research results, researchers kept a reflectivity diary for continuous self-censorship, reflection, and bracketing. Initially, given the severity of the pandemic, the researchers noted that they had an early preconception that patients with COVID-19 in Wuhan would have negative psychological experience. However, as the interviews progressed, the emerging interview data challenged this preconception.

### Ethical consideration

The current study followed the guidelines of the Declaration of Helsinki of 1975, revised in 2013, as well as conforming to principles of medical research ethics, and was granted ethical approval by the Ethics Committees of Shenzhen Hospital, Southern Medical University on March 13th, 2020 (No. NYSZYYEC20200007). Before the interview, subjects were informed of the purpose, methodology, and voluntary nature of the study. Written informed consent was obtained from all participants to participate in the research, and for the interview to be audiotaped. Each interview was conducted in a quiet and separate ward with just the participant and the interviewer. Confidentiality was ensured by removing identifiable information from the transcripts and using serial numbers instead of names (e.g., P1, P2).

## Results

**Fig. 1:**
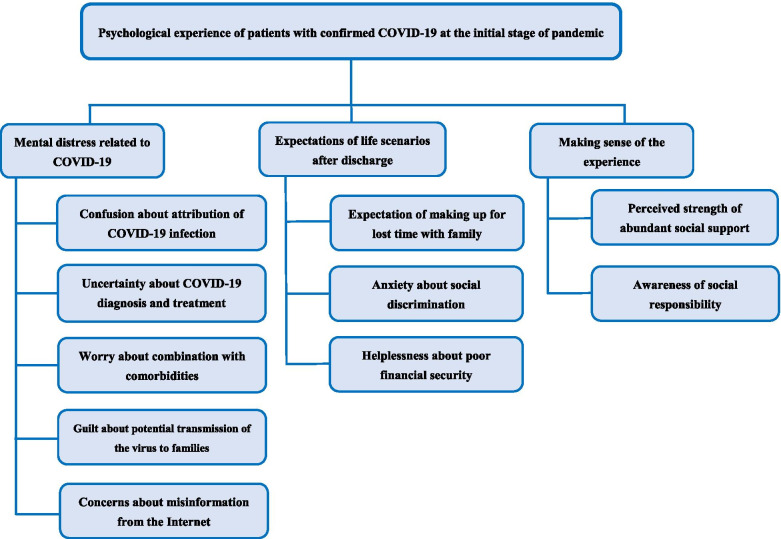
Key themes and subthemes of psychological experience of patients with confirmed COVID-19 at the initial stage of pandemic

Three key themes emerged from the analysis of interviews: mental distress related to COVID-19, expectations of life scenarios after discharge, and making sense of the experience. All of the themes and subthemes are summarized in Fig. 1.

### Theme 1: mental distress related to COVID-19

#### Confusion about attribution of COVID-19 infection

The first sub-theme within the first theme category was *confusion about attribution of COVID-19 infection*. Participants reported feeling doubt about the reasons they contracted the disease. As an emerging infectious disease at the initial stage of the pandemic with a highly contagious nature, transmission patterns of COVID-19 remain to be poorly understood. Given their good physical condition, avoidance of hazardous behaviors and practice of preventive measures, most patients did not understand why they contracted COVID-19. Participants expressed feeling shock and doubt when they were diagnosed with COVID-19:

*“... I sold subsidiary food in a supermarket. My job had nothing to do with seafood products. What’s more, there were very few people in the supermarket during the Lunar New Year holidays...Don't you think it's strange? I felt very confused...my house was well disinfected. I don’t know what went wrong.”*

*(P3, female)*

*“Before I contracted COVID-19, my body was in a generally good condition, except for surgery 10 years ago. I had not taken any medication for a very long time. I have no idea how I became infected.”*

*(P4, female)*

COVID-19 is featured by a relatively high rate of re-detectable positive result after negative detection of virus nucleic acid test on nasopharyngeal swabs. Patients described that they felt puzzled about having a further positive test. They wondered why this strange phenomenon occurred:

“I received four virus nucleic acid tests on nasopharyngeal swab samples. The first three consecutive tests were SARS-CoV-2 negative but the fourth test was positive again. Why?”

*(P1, male)*

#### Uncertainty about COVID-19 diagnosis and treatment

The second sub-theme identified was *uncertainty about COVID-19 diagnosis and treatment*. Many of the patients described psychological distress due to a sense of uncertainty. Varieties of unknown aspects of COVID-19 currently still exist at the initial stage of the pandemic. In addition, patients generally lacked medical knowledge about the disease or relevant medications. Patients mentioned that they were uncertain about the therapeutic effects of medication, and were deeply concerned about drug discontinuation after being discharged. Participants reported that their uncertainty was further aggravated by the high rate of re-detectable positive COVID-19 tests after earlier negative results:

*“...If I received gamma globulin injections, virus nucleic acid tests on nasopharyngeal swab samples turned negative; if I did not, my tests turned positive again. So what if the tests turn positive again after being discharged?”*

*(P2, female)*

In addition, patients with COVID-19 pointed out that virus nucleic acid tests were less stable and precise. They lacked information about the working principles of virus nucleic acid tests. As well, patients expressed doubts regarding the sensitivity and specificity of the test:

*“I feel very confused about the results of the virus nucleic acid test. I was told that the accuracy of the test is 60%. However, in fact, the accuracy was only 40% in my opinion... I am perplexed that half of the tests showed false negatives.”*

*(P11, male)*

#### Worry about combination with comorbidities

The third sub-theme within the first theme category was *worry about combination with comorbidities*. The severity of COVID-19 varies among patients, and elderly people and those with comorbidities are more likely to develop severe illness. Participants with comorbidities noted that they were particularly worried about interactions between COVID-19 and their comorbidities:

“... I have hypertension. Will COVID-19 aggravate my comorbidities? I always feel stuffy in this ward...“

*(P1, male)*

#### Guilt about potential transmission of the virus to families

The fourth sub-theme within the first theme category was *guilt about potential transmission of the virus to families*. Due to the extremely high infection rate of COVID-19, patients perceived themselves as a source of danger that threatened the health of family members. Patients were worried about spreading the virus to their families, particularly their children and felt guilty about the possibility of transmission:

*“... And knowing that my wife has also contracted this disease, I blame myself for living with my wife and child before the quarantine... it is a comfort for me that my child has not contracted COVID-19. Fortunately, it was the adult and not the child who contracted COVID-19.”*

*(P10, male)*

#### Concerns about misinformation from the internet

The fifth sub-theme within the first theme category was *concerns about misinformation from the Internet*. Emerging Internet technology has greatly facilitated information transmission. In this case, both accurate and false information can spread more conveniently and rapidly than ever. The management of information from the Internet is highly challenging. Patients expressed that they were psychologically distressed by misinformation from the Internet:

*“I have joined many WeChat Groups duringstaying in the hospital. Many posts contain information, which is true or false, positive or negative. Although this information does not necessarily change my overall mindset, it does influence me.”*

*(P6, male)*

*“... I have witnessed the whole process from contracting COVID-19, to the building of Fangcang shelter hospitals and to the present. In my opinion, COVID-19 isn’t as horrible as is usually believed. The reports on the Internet seem to exaggerate it.“*

*(P12, female)*

### Theme 2: expectations of life scenarios after discharge

#### Expectation of making up for lost time with family

The first sub-theme within the second theme category was *expectation of making up lost time with family*. Patients typically reported playing multiple social roles in their families, such as parents and spouses. However, the pandemic of COVID-19 meant that patients could no longer take on their usual family responsibilities, such as childcare. Patients were motivated to make up for lost time during quarantine:

*“... I will make up for lost time after I am discharged and released from quarantine. I have spent two months in hospital without going out...It is a gorgeous season of the year with spring blossoms and I want to make up for everything lost in the past two months.”*

*(P12, female)*

#### Anxiety about social discrimination

The second sub-theme within the second theme category was *anxiety about social discrimination*. COVID-19 has attracted global attention with the large-scale pandemic. Patients reported that they were anxious about social discrimination after being discharged, feeling that the general public may discriminate against them because of the risk of human-to-human transmission. Patients reported being faced with tremendous pressure and challenges even after being discharged to resume normal life:

*“...when I am still in the hospital, the WeChat Group of my residential community has a post that contains “...what can we do if the patients are discharged from the hospital and returned to the community? There is a chance of being infected if we get in touch with them when going out for walking or shopping”... after seeing that post, I don’t know what to do after going back...(Sigh)”*

*(P3, female)*

*“I think there will be some social discrimination. The world outside certainly discriminates against us to some degree... that poses an intangible pressure upon us.”*

*(P13, female)*

#### Helplessness about poor financial security

The third sub-theme within the second theme category was *helplessness about poor financial security*. The pandemic of COVID-19 posed a severe challenge to the general public’s daily routine and socioeconomic systems. Although all medical expenses of patients with COVID-19 were covered by central and local financial institutions, patients reported that they were still faced with significant economic pressure. Patients expressed that their lives after being discharged were highly uncertain and that they had to handle the challenge by themselves:

*“... I want to go out to work. I want to make money. I need to live on my own. I have not retired yet...“*

*(P1, male)*

*“Working in the supermarket was tiring... I guess I cannot stand the hardships anymore, and I don't know how to make a living after I am discharged...without medical insurance in Wuhan, I do not have access to support in case of illness. I have to handle this all by myself.”*

*(P3, female)*

### Theme 3: making sense of the experience

#### Perceived strength of abundant social support

The first sub-theme within the third theme category was *perceived strength of abundant social support*. Although the patients were isolated at the hospital, they felt the warmth of affection and friendship. They reported that they received social support from a variety of sources, including HCWs, families and friends, both physically and spiritually, to combat the virus. Patients expressed that they were grateful to frontline HCWs for their efforts to take great risks to provide care to them:

*“ ..your efforts make me feel warm (Smile). Care of HCWs is the most selfless and relieves my anxiety”*

*(P5, female)*

*“... before the pandemic, I did not pay enough attention to these small things. However, after I contracted COVID-19, I feel that it is a blessing to have all these friends and relatives who give me so much support.”*

*(P7, male)*

Apart from families and friends, patients with COVID-19 also appreciated government staffs and reported that the local and national governments provided generous support and strong backing at the macroscopic scale:

*“We might have been dead if without the governments’ efforts. The medical rescue team has been providing selfless service for us under the government policy [patients with COVID-19], even to the extent of ignoring themselves. I really respect them [frontline HCWs]. ”*

*(P3, female)*

#### Awareness of social responsibility

The second sub-theme within the third theme category was *awareness of social responsibility*. Hospitalized patients with COVID-19 experienced severe psychological stress. Despite the desire to return to their normal lives, patients kept in mind the social responsibility to protect their families and other people. Most patients reported they were willing to continue self-quarantine until they were no longer infectious:

*“I will continue self-quarantine to be responsible for myself, family and society.”*

*(P9, male)*

During the COVID-19 crisis, patients reported receiving intense support from family, friends, HCWs and their country. This further motivated the awareness of social responsibility among COVID-19 patients. Patients expressed their appreciation and desire to give back to society:

*“... after I recover from COVID-19, I will learn from the fearless and selfless medical workers. I want to make my contribution to the country, fulfill my full ability, and give back to society.”*

*(P8, male)*

## Discussion

The current study qualitatively investigated the psychological experience of patients with confirmed COVID-19 in Wuhan, the area hit hardest by COVID-19 in China at the initial stage of pandemic, revealing that patients were distressed by the pandemic and experienced severe psychological impacts regarding COVID-19 and their life scenarios after discharge. Despite this, patients made sense of the experience with perceived strength of abundant social support and awareness of social responsibility. The findings of this qualitative study contribute to a comprehensive understanding the psychological impacts of the disease, both negative and positive, which is important for further addressing psychological problems and relieving psychological stress.

### Mental distress related to COVID-19

The first psychological experience of patients with COVID-19 was referred to mental distress related to COVID-19. Pandemic-related mental distress was one of the main psychosocial challenges of COVID-19 [21]. As voices from COVID-19 frontline nurses, there were multiple forms of negative experience about COVID-19 among patients, including confusion, uncertainty, worry, guilty and concerns [22, 23]. The majority of mental distress could be attributed to high infectivity of COVID-19, poor knowledge and no effective anti-viral therapeutics that specifically targets COVID-19 at the initial stage of pandemic [24, 25]. These facts led to patients’ unsatisfactory condition, great concerns about the disease and infecting families. Additionally, patients with comorbidities were more emotionally troubled than others as observed in other study [26].

Apart from that, a significant difference from the SARS epidemic in 2003 is the prominent extent and speed of information flow [27]. The present study indicated that patients with COVID-19 were troubled by the misinformation from the Internet. On the one hand, as reported in a quantitative study by Zhong et al. [28], use of social media was a significant predictor of depression and secondary trauma. Further, excessive use of social media could lead to more severe symptoms of depression and secondary trauma. On the other hand, there is a mass of misinformation disseminated via commonly used social media platforms, including disease statistics, preventive measures, and others [29]. This condition can be called “coronavirus infodemic” as termed by WHO [30] and can lead to psychological turmoil, such as anxiety, panic and traumatic reactions [31, 32]. Therefore, addressing the issues of coronavirus infodemic are helpful to moderate distress in patients with COVID-19.

### Expectations of life scenarios after discharge

The second psychological experience of patients with COVID-19 is related to expectations of life scenarios after discharge. As observed in our study, they expected to make up for the lost time with family during hospitalization. Except for the expectations of fulfilling their family responsibilities after discharge, concerns about social discrimination and poor financial security were also identified. This can be regarded as negative expectations, that less advantageous consequences give rise to an emotional state of disappointment [33]. Anxiety about social discrimination can be called COVID-19-related stigma, which is a crucial dimension in conceptual framework of health-related quality of life in hospitalized COVID-19 survivors [34]. The occurrence of stigma is largely caused by absence of good education about the novel epidemic [35]. It can cause excess psychosocial barriers and tensions [36] and is also an obstacle to health-seeking behavior and then delaying COVID-19 diagnosis and treatment [22, 37]. Hence, it is important to prevent the occurrence of COVID-19-related stigma to facilitate the reintegration into community of patients after being charged. Besides, poor financial security also greatly contributed to reduced mental health. It was in line with the study conducted during recovery following the outbreak of SARS [38], which reported that economic concerns were the most common psychological disorder. However, migrant workers confirmed with COVID-19 in Singapore had not expressed concerns about subsequent financial problems [39]. One possible reason of the difference was that migrant workers in Singapore could continuously receive wages during quarantine. This may provide a guide of policy-making in the future.

### Making sense of the experience

The third category psychological experience of patients with COVID-19 included perceived strength of abundant social support and awareness of social responsibility, that providing a meaning for the extraordinary experience. Although the patients experienced threats to their mental health, in response to COVID-19 pandemic, they also achieved “stress related growth”. Social support and social responsibility are mainly two protective factors of mental health during psychological adaptation process [40]. Specifically, the social support is external factor. Receiving support effect on increasing positive outcomes in patients’ relaxation and recovery [41]. Support of the families and HCWs were also reported in other regions [42], while support of government was a country-specific result. The positive feelings towards government need to be situated within the Chinese contexts. Moreover, the awareness of social responsibility is internal factor, which is highlighted in the Chinese collectivism-oriented culture and Confucian tradition of morality [43]. A person is taught altruism and responsibility for others in the traditional Chinese culture. For example, the most commonly expressed sentiment of patients was willingness to continue to self-quarantine after discharge to avoid transmitting the virus to others. The same theme also emerged in a study involving interviews with frontline HCWs [44].While in other countries, individuals’ behavior is profoundly influenced by religious factors, such as Singapore, Iran [39, 41].

### Strengths and limitations

This was a unique study focused on elucidating the psychological experience of patients with confirmed COVID-19 in Wuhan, the area in China hit hardest by COVID-19, at the initial stage of the pandemic. Currently, few qualitative studies were performed and limited to a specific group, such as survivors throughout COVID-19 crisis [45], patients involved in the transmission of family cluster [35]. The results extended current knowledge about psychological experience among patients with COVID-19 especially in the context of China and may inform the measures taken worldwide to cope with these psychological issues. Furthermore, the phenomenological approach in qualitative research, aiming to interpret the essential meaning of a lived experience, enabled the in-depth gathering of rich experience in our qualitative study [16]. Accordingly, inductive thematic analysis through a line-by-line open coding and respecting the interviewees’ voice is suitable for a phenomenological approach [19].

However, our study also had several limitations that should be considered. First, due to the urgency at the initial stage of the pandemic, the inclusion criteria of without language barriers was subjectively judged by researchers and there were no more time, funds and other resouces to support performing the more objective and complex tests. As Ayres stated that sampling and data collection need to be at the appropriate level of complexity for the design while keeping the study feasible in its use of resources at the same time [46]. Besides, the interviewers were frontline nurses in an intensive condition, that may influence the length of interview time. The indepth of interviews was limited because of the short average length of interview time. Nevertheless, the observation and field notes were used to concurrently collect information during interview as an extra data source to supplement the interview data. Second, the research context was in a COVID-19-designated hospital in Wuhan. Due to the difference of hospital environment, regions and countries, these results may vary from each other. Third, the sample only represented married, emotionally stable and patients with common-type COVID-19 from the area hardest hit by COVID-19 in China, limiting the generalizability of the current findings to COVID-19 patients as a whole. Forth, purposive sampling and coders’ bias are heavily dependent on the researchers’ judgment, which is an inherited disadvantage of qualitative method. Although we adopted strategies to minimize reflexivity and maximize reflection, the present findings should be further validated by supplementary quantitative methodology in the future.

### Practical implications and future research

The present study highlighted that patients with COVID-19 need to receive not only medical care to cure physical illness but also psychological interventions. Similar to the current results, the major sources of psychosocial vulnerabilities, including medical comorbidities, stigmatization and socio-economic difficulties [47], misinformation on the Internet [29] have also been reported in other countries. To help reduce patients’ confusion, uncertainty and worry, HCWs are expected to provide patient education on features and available therapeutic regimens of COVID-19 as well as their current disease progression to improve their illness perception and relieve their mental distress by promoting patient empowerment [48, 49]. Furthermore, taking every available measure to promote dissemination of accurate information and guarantee employment should be a priority for public health authorities and local governments. Positive emotion associated with making sense of the experience presented in our study was a new and attractive finding with important implications. Positive emotions play a critical role in adjustment and rehabilitation of psychological trauma [50, 51]. Due to lack of professional psychologists under the strict infection prevention measures, frontline HCWs could take up the responsibility of providing targeted psychological interventions to patients, to reinforce the positive emotions and relieve the negative emotions of patients [52].

Because of the exploratory nature of this study, quantitative assessments and follow-up should be employed to in future studies to confirm the current findings. Longitudinal qualitative studies in a large scale are also encouraged to explore the psychological trajectory of patients with COVID-19. These findings can provide useful guidance and direction for practitioners and policymakers developing approaches for relieving mental distress in patients affected by COVID-19.

## Conclusions

The current study provided a comprehensive understanding of psychological experience of patients with COVID-19 at the initial stage of the pandemic through in-depth interview using a phenomenological approach. We found that patients had complex psychological experience during the COVID-19 crisis, with the manifestations of mental distress related to COVID-19, expectations of life scenarios after discharge, and making sense of the experience. To illuminate the neglected positive psychology experience of patients with COVID-19, future research should deeply and holistically analyze the psychology experience of patients. Future research on how to provide psychological intervention to reinforce the positive emotions, relieve the negative emotions of patients and safeguard their wellbeing is also warranted.

## Data Availability

The datasets used and/or analysed during the current study are available from the corresponding author on reasonable request.

## Abbreviations

COVID-19: Coronavirus disease 2019
SARS-CoV-2: Severe acute respiratory syndrome coronavirus 2
SARS: Severe acute respiratory syndrome
HCWs: Health care workers
WHO: World health organization
